# Italian Validation and Psychometric Properties of the New Work Values Scale

**DOI:** 10.3390/ejihpe15030028

**Published:** 2025-02-25

**Authors:** Lavinia Cicero, Carlotta Catania, Adriano Russo, Andrea Zammitti, Angela Russo, Giuseppe Santisi

**Affiliations:** 1 Department of Theoretical and Applied Sciences, e-Campus University, 22060 Novedrate, Italy; lavinia.cicero@uniecampus.it (L.C.); hadrian.russo7@gmail.com (A.R.); 2 Department of Educational Sciences, University of Catania, 95124 Catania, Italy; carlotta.catania98@gmail.com (C.C.); angela.russo18@unibo.it (A.R.); gsantisi@unict.it (G.S.); 3 Department of Psychology, University of Bologna, 47521 Cesena, Italy

**Keywords:** work values, work orientations, career, validation, psychometric properties

## Abstract

The construction of a fulfilling career and the people management processes within organizations, like the selection of personnel, require a multidisciplinary approach that takes into account psychological, social, and cultural factors. Various concepts have been suggested to explain work motivations and organizational outcomes, including work values. Work values can encompass individual preferences, as well as moral standards and social norms. This broad definition has led to a variety of work value measurement instruments. One brief and cutting-edge measure that integrates different approaches is the New Work Values Scale (NWVS). The aim of this study was to validate the Italian form of this measure (NWVS-I). A sample of 397 Italian adults from 19 to 66 years of age (M = 30.78, SD = 13.38) participated in the study and completed both the New Work Values Scale—Italian form (NWVS-I) and the Portraits Value Questionnaire (PVQ). First, we evaluated the structure of the New Work Values Scale—Italian form (NWVS-I) through confirmatory factor analysis (CFA), followed by a concurrent validity analysis correlating the dimensions of the New Work Values Scale—Italian form (NWVS-I) with those assessed by the Portraits Value Questionnaire (PVQ). We also tested gender invariance. The results confirmed the factor structure of the scale and its validity in the Italian context. The New Work Values Scale—Italian form (NWVS-I) is a useful measure in understanding the work values of individuals in the Italian context. This measure can be used for a wide range of applications, contributing to promoting greater awareness of one’s values and facilitating career choices, personnel selection, and people management aligned with a vision of sustainable organizational development.

## 1. Introduction

Current times are deeply marked by a profound revolution due to technological, demographic, and environmental changes that require a reevaluation of organizational psychology’s current approaches to work design and redesign (Fraccaroli et al., 2024). The liquid modernity described by Bauman (2013) is devoid of ideals of stability and favors ambiguity, temporariness, and uncertainty. These conditions have had a major impact on the economic market and the world of work. Constant change, unpredictability, and confusion can distance us from the aspirations and values that guide our actions and professional choices (Blustein et al., 2019). Since the economic crisis of 2008 and the technological evolution over the last twenty years, there has been a progressive change in the structure and quality of work worldwide and internationally. In response, participatory human resource actions are essential to fostering workers’ willingness to adapt to these changes (Zammitti et al., 2022). In particular, the case of Italy stands out from within the complex European picture. Historically characterized by heterogeneity and marked differences in industrialization and migration flows between the northern and southern regions (Bentivogli et al., 2019), in recent years, Italy has experienced lower employment rates and job quality than the European norm (Camussi et al., 2021). These findings describe a characterization of the world of work that is opposite to what younger workers, in particular, seek, namely work–life balance, work stability, and financial independence (Bosch & Hernández, 2020; Sousa, 2021). The COVID-2019 pandemic has intensified the problems of a labor market already burdened by the difficulties of organizations in recruiting and hiring workers to find job positions in line with their desires and aspirations, thus worsening labor and social inequalities (Ramskogler, 2022).

Recently, researchers and practitioners in the psychosocial area have focused on the study of the role that psychosocial factors play in promoting better adaptation between people and the environment, taking an interest in identifying the resources involved in facing the challenges of recent economic and social changes, especially in value contexts such as work (Magnano et al., 2024). In the context of promoting both individual and organizational well-being, values play a central role because having clear values can help improve well-being and performance as they represent “the personal strengths or qualities a person most wants to express in his or her goals and daily patterns of action” (Flaxman et al., 2013, p. 85). In other words, being clear about one’s values can contribute to the preventive and promotional enhancement of people’s psychosocial capabilities and resources in the work context as well. This is due to the fact that values shape motivation, goal-setting, and behavioral regulation. Through the Self-Determination Theory (Deci & Ryan, 2000), it has been contended that when work is aligned with personal values, it facilitates autonomy, purpose, and engagement that results in better well-being and performance. Similarly, Schwartz’s Value Theory (Schwartz, 1999) describes how values impact decision-making and flexibility, resulting in resilience and organizational commitment. This, in work-related contexts, is reflected through greater job satisfaction, proactive behavior, and greater alignment with one’s professional calling.

Work values encompass a broad range of evaluative standards related to work or the working environment, encompassing individual preferences, moral principles, and societal norms (Dose, 1997). Work values are a rather broad field of study that encompasses multiple contributions, including those concerning how they have changed over time in different generations. The well-known model of Schwartz and colleagues (2001) sees work values as beliefs pertaining to desirable end-states or behaviors and can be divided into four types: intrinsic (personal growth, autonomy, interest, and creativity), extrinsic (pay and security), social (contact with people and contribution to society), and power (prestige, authority, influence) (Schwartz, 1999; Ros et al., 1999). According to Fossen and Vredenburgh (2014), work values are necessary for individuals to find meaning, satisfaction, and well-being in a given job role. Several authors have expanded this conceptualization, explaining how people’s orientations toward work relate to prestige, status, fulfillment, and social connections (Pitacho et al., 2019; Willner et al., 2019). According to Höge (2011), the new reality in which the modern worker moves makes him or her an “entreployee”, capable of working with flexibility, autonomy, development, opportunities, adaptation, and new challenges. Another key contribution relates to the framework of Self-Determination Theory (Deci et al., 2017); according to this theory, workers seek to satisfy their needs related to competence, autonomy, and relationships: by supporting them, organizations promote the development and success of both employees and organizations (Rigby & Ryan, 2018; Forner et al., 2020). Investigating generational differences in work-related values, Reis and Braga (2016) state that younger generations place greater importance on work values related to instrumental factors, such as economic stability. In addition, there has recently been a greater emphasis on values related to sustainable organizational development (Stiglbauer et al., 2022). For example, corporate social responsibility (CSR) and inclusion are key factors for improving employer branding. Indeed, corporate social responsibility significantly influences organizations by improving their reputation, fostering employees’ trust and organizational support, and leading to increased job performance and organizational citizenship behavior (Trivellas et al., 2019; Kim & Kim, 2020; Mauk & Halek, 2022; Bharadwaj, 2023). An inclusive environment fosters an employee’s sense of belonging and self-esteem, increases organizational commitment, and reduces turnover rates, leading to improved productivity and organizational well-being (Laskin & Kresic, 2021; Kasih & Ruslaini, 2024). To be effective, it is crucial that these aspects are aligned with the different identities and preferences of potential employees so that organizations can improve their attractiveness and effectiveness in recruiting top talent (Buzzao & Rizzi, 2024).

Building on these contributions, and on the relative evolution of work values in the 21st century, Stiglbauer and colleagues (2022) developed a tool capable of synthesizing all the theoretical content presented so far and investigating instrumental and symbolic dimensions of work values in a simple and fast way to improve employer branding strategies, i.e., the set of instrumental attributes that influence the attractiveness of an organization in the eyes of candidates and represent a fundamental antecedent for attracting and retaining talent (Backhaus & Tikoo, 2004; Theurer et al., 2018; Reis et al., 2021). The New Work Values Scale (NWVS; Stiglbauer et al., 2022) investigates 14 dimensions of work values, including readiness for change (a preference of new opportunities), corporate social responsibility (the search for organizations aimed at economic, legal, ethical and philanthropic responsibility), inclusion (the search for organizations with policies of fairness and equality), job security (the strong need for job stability), participation (the search for contexts in which everyone can contribute), clarity (the need for consistently established structures and guidelines), flexibility (the search for a work–life balance) development (the need to enhance professional competences and skills), career (new career opportunities perceived as important), money (the importance of monetary rewards), stimulation (the need for variety and challenge), autonomy (the possibility to design and perform one’s work freely), meaning (the need for meaningful and socially useful work), and the ability to relate (the development of a network of relationships in the workplace). These values can be grouped into three areas as follows: sustainable organizational development (including readiness for change, corporate social responsibility, and inclusion), basic needs (including job security, participation, clarity, and flexibility), and individual motivators (including money, career, development, stimulation, autonomy, meaning, and relating). This classification provides a clear and structured overview of different work motivations. Each value represents a critical aspect that can guide career decisions, engagement, and professional satisfaction. Organizations can use this taxonomy to align their values and strategies with employee expectations, thereby improving recruitment, motivation, and retention. Additionally, in a short period of time, this scale makes it possible to assess a wide range of values that may be the key to increasing the attractiveness of the company in the eyes of external professionals. Congruence between organization and employee values contributes to job satisfaction and a lower risk of turnover and commitment to the company (Arieli et al., 2020; Busque-Carrier et al., 2021; Anglim et al., 2022).

Given the absence of a broad and up-to-date scale, albeit a short one, in the Italian context, the aim of the present study is to confirm the factor structure of and, therefore, to evaluate the psychometric properties of the Italian form of the New Work Values Scale (NWVS; Stiglbauer et al., 2022), so as to offer the academic world and organizations important hints for orienting HRM policies in the contemporary panorama. Specifically, we aim to evaluate the structure of the New Work Values Scale (NWVS) through confirmatory factor analysis (CFA), followed by a concurrent validity analysis correlating the dimensions of the New Work Values Scale (NWVS) with those assessed by the Portraits Value Questionnaire (PVQ).

While existing work value instruments (Super, 1970; Rounds et al., 1981; Nord et al., 1988) can assess core dimensions such as intrinsic and extrinsic motivations, the NWVS includes both traditional and emerging values, including corporate social responsibility, diversity, and adaptability. Unlike more generic frameworks like the PVQ (Schwartz et al., 2001), which assesses general value orientations, the NWVS specifically targets work values in a way that reflects the realities of contemporary workforce expectations. It is designed to allow for a concise yet comprehensive assessment, making it a useful tool for career guidance, HR decision-making, and employer branding activities.

Demand for an Italian form of the NWVS is motivated by the unique features of the Italian labor market, where topics such as labor security, work–life balance, and career continuity are still dominant issues (Camussi et al., 2021). Meanwhile, constructs of corporate social responsibility values, diversity, and flexibility are gaining increasing relevance, especially for the young generation (Reis & Braga, 2016). Compared to more general value measures like the PVQ, the NWVS allows for a more precise examination of work-related motivations, offering results valuable for research and also for applied purposes in HRM and career counseling.

## 2. Materials and Methods

### 2.1. Participants and Procedure

Determining the appropriate sample size or statistical power is a critical step in research design (Tabachnick & Fidell, 2013). One widely used method for estimating sample size is the sample-to-item ratio, which calculates the required sample size based on the number of items in the study. According to the guidelines established, this ratio should not fall below 5:1 (Hu & Bentler, 1999; Brown, 2015; Kline, 2016). Moreover, to account for the number of dimensions in the CFA for both measurement instruments involved in this study (see below), the sample size was calculated using Soper’s Calculator (Soper, 2023). Assuming an acceptable alpha error of 0.05, aiming for 95% power, and considering 18 latent variables and 54 observed variables, based on an effect size of 0.30, the minimum required sample size was determined to be 308.

Once the minimum sample size was determined and the research protocol was finalized, participants were invited to take part in the study through voluntary recruitment. Data collection was conducted using an online questionnaire hosted on a digital platform. We used a convenience sampling approach, where participants were selected from the researchers’ personal and professional networks. A snowballing procedure was then employed, asking participants to refer others who met the study criteria. Responses were automatically centralized into an online spreadsheet, ensuring efficient and accurate data management. Participants were required to provide explicit consent for their participation and the anonymous processing of their data in accordance with ethical research guidelines.

The inclusion criteria for participation were as follows: (1) being a native Italian speaker, (2) being of legal age (18 years or older), and (3) providing informed consent for data collection and processing.

The final sample consisted of 397 participants: 249 females, 144 males, 1 non-binary individual, and 3 individuals who preferred not to disclose their gender identity. Participants ranged in age from 19 to 66 years (M = 30.78, SD = 13.38). In terms of educational background, the majority (262 participants) held an upper secondary school diploma. Of the remaining participants, 101 had obtained a bachelor’s or master’s degree, 11 had a doctoral degree or equivalent, 9 participants had a middle school diploma, and 3 held a primary school diploma. Regarding employment status, 183 participants were students, while the others were employed (171) or unemployed/seeking their first job (43).

### 2.2. Ethical Considerations

This study adhered to the ethical principles outlined in the Ethical Code of the Italian Association of Psychology (AIP). Additionally, it was submitted to the Ethics Committee of the University of Catania for review. The committee approved the study, confirming that it posed no health risks to participants (approval number: Ierb-Edunict-2024.06.03/07).

### 2.3. Measure

The questionnaire used for data collection included the following sections and measures:

**Sociodemographic data.** The initial section collected sociodemographic information such as gender, age, educational background, and employment status.

**New Work Values Scale** (NWVS; Stiglbauer et al., 2022). The New Work Values Scale (NWVS) is a psychometric instrument designed to assess individuals’ values and preferences in the modern workplace. This scale includes two items for each dimension: *readiness for change* (individuals who prefer organizations that do not persist in old practices, but are open to innovations, consider change as an opportunity, and are ready to implement new ideas); *corporate social responsibility* (individuals looking for organizations that are well aware and take care of their economic, legal, ethical, and philanthropic responsibility); *inclusion* (individuals who want organizations to especially care about the fair and respectful treatment of all members and fight discrimination by all means); *job security* (individuals with a strong need for a secure workplace); *participation* (those who prefer a job where hierarchies are flat and everyone is welcome to contribute his or her own opinion and ideas); *clarity* (individuals who need structure, rules, and guidelines that provide stability, consistency, and orientation); *flexibility* (those who want their job not to interfere with their personal lives); *money* (individuals mostly motivated by monetary rewards); *career* (those who consider career development opportunities very important to them); *development* (individuals who want to give their best and to, therefore, further develop their professional knowledge, skills, and competencies); *stimulation* (those who have a strong need for variety, challenges, and do not have much going on in their job); *autonomy* (individuals who want to design and do their work self-directedly); *meaning* (those who need their work to be meaningful and serve a collective purpose); *and relating* (individuals who place great emphasis on good social relationships at work). Each dimension is measured using a specific Likert scale ranging from 1 (strongly disagree) to 5 (strongly agree).

The cultural adaptation of the NWVS followed the procedure outlined by Beaton et al. (Beaton et al., 2000). Two researchers independently translated the items and instructions. Their translations were then compared, and a consensus was reached on a finalized version of the scale. This final version was subsequently back-translated into English by a native speaker to identify and implement any necessary refinements.

The version used in this study is provided in Appendix A.

**Portrait Values Questionnaire** (PVQ; Schwartz et al., 2001; Capanna et al., 2005). The PVQ consists of a brief “portrait” of a typical person, highlighting their goals, aspirations, or desires. Participants were asked to rate how similar they perceived the described person to themselves, using a six-point scale ranging from “*very much like me*” to “*not like me at all*”. The PVQ allows the calculation of scores for the four value dimensions theorized by Schwartz (1992): *openness to change* (10 items), *conservatism* (13 items), *self-enhancement* (7 items), and *self-transcendence* (10 items). In this study, Cronbach’s alpha was 0.76, 0.80, 0.86, and 0.80, respectively.

### 2.4. Data Analysis

First, all descriptive statistics for the items were calculated as follows: the mean, standard deviation, skewness, and kurtosis. According to Hair et al. (2010) and Byrne (2010), data can be considered normal if skewness falls within the range of −2 to +2 and kurtosis is within the range of −7 to +7. Thus, the data were analyzed using the original Likert scale metric (Robitzsch, 2023).

Since the aim of this study was to confirm the factor structure of the instrument in Italy, we proceeded directly with a confirmatory factor analysis (CFA). CFA was conducted using AMOS 22.0.0 to compare and identify the best-fitting model. Three models were tested as follows: (1) a model with 14 factors; (2) a model with 14 factors and 3 high-order factors (sustainable organizational development, basic needs, and individual motivators); and (3) a single-factor model. It was hypothesized that the 14-factor model would demonstrate superior fit indices compared to the others.

Model fit was evaluated using several criteria: the chi-squared to degrees of freedom ratio, which should fall between 1 and 3; the Comparative Fit Index (CFI), which should exceed 0.90 (Bentler, 1990); and the Root Mean Square Error of Approximation (RMSEA) and Standardized Root Mean Square Residual (SRMR), both of which should be less than 0.08 (Steiger, 1990). To compare the models, the Akaike Information Criterion (AIC) was utilized, with lower values indicating better model fit (Burnham & Anderson, 2004). Differences in AIC greater than two were considered statistically significant, highlighting differences between the models (Jöreskog & Sörbom, 1993).

To assess the internal consistency of the scale, we computed both Cronbach’s Alpha (α) and Hancock’s H. Cronbach’s Alpha (Cronbach, 1951), which is one of the most widely used measures of internal reliability. We also computed Hancock’s H (Hancock & Mueller, 2001), which provides a more robust measure of construct reliability in confirmatory factor analysis (CFA). Both indices were computed for each factor of the scale. The results were compared to ensure consistency in the assessment of internal reliability.

The average variance extracted (AVE) was computed to measure the proportion of variance in observed variables accounted for by their respective latent constructs, following the recommended threshold of at least 0.50 (Hair et al., 2010). High and statistically significant correlations among scales may indicate potential multicollinearity issues. Discriminant validity assesses a latent construct’s ability to be distinct from other constructs in the model. It was calculated with the square roots of average variance extracted (AVE); if the results were superior to correlations between constructs, there was good discriminant validity (Fornell & Larcker, 1981).

Concurrent validity was evaluated by examining the correlation with a comparable measure of values. Specifically, a positive correlation with other values was expected. The results were interpreted as follows: low correlation (r = 0.10–0.29), moderate correlation (0.30–0.49), and high correlation (r > 0.50) (Cohen, 1988).

To test for gender differences between males and females, we used the t-test, accompanying this analysis with Cohen’s d. Cohen’s d is an additional metric of effect size. The following guidelines can be used to interpret this index: a small effect (<0.2), medium effect (<0.5), and large effect (<0.8) (Cohen, 2013; Magnusson, 2021).

To evaluate gender invariance, a multiple-group CFA was conducted, and the best-fitting model was selected. For this analysis, the sample was divided into two groups based on gender, considering only those who identified as male or female. The following types of invariances were assessed: (1) configural invariance, ensuring equivalence in the model structure; (2) metric (weak) invariance, testing the equivalence of factor loadings; (3) scalar (strong) invariance, examining the equivalence of item intercepts or thresholds; and (4) residual invariance, examining the equivalence of item residuals or unique variances. To confirm metric and scalar invariance, changes in fit indices throughout the process were required to remain within Δ < 0.01 for CFI and Δ < 0.015 for RMSEA (Putnick & Bornstein, 2016).

## 3. Results

Table 1 provides descriptive statistics (the mean, standard deviation, skewness, and kurtosis) for the 28 items of the NWVS-I scale, divided into 14 dimensions. The mean scores range from 3.37 (MON 2—“Money”) to 4.76 (FLE 2—“Flexibility”). Most items show negative skewness, with values ranging from −1.92 (FLE 2—“Flexibility”) to −0.24 (AUT 2—“Autonomy”). Kurtosis values range from −0.72 (AUT 2—“Autonomy”) to 2.88 (FLE 2—“Flexibility”). In line with the guidelines mentioned, the items appear to be normally distributed. A table in which we provide item–category frequencies for all items of NWVS-I is available in the Supplementary Material.

Table 2 presents the results of three fit models evaluated for the structure of the NWVS-I scale. Model 1 (14 factors) demonstrated excellent fit and the lowest AIC value, indicating that this model is the best among the three. The results suggest that the 14-factor model adequately represents the data, with fit indices close to ideal values. Model 2 (14 factors with 3 high-order factors) showed an acceptable fit but had a CFI below the desired threshold and a higher AIC value than Model 1, suggesting a loss in model quality. Thus, this model does not fit the data as well as the first model. Model 3 (a single factor) exhibited poor fit, as evidenced by the fit indices and a significantly higher AIC value compared to Models 1 and 2. This clearly indicates that the single-factor model does not adequately represent the data, and the hypothesis of a single factor is not supported by the data.

In conclusion, Model 1 (14 factors) emerges as the best model, with excellent fit indices and the lowest AIC, supporting a multidimensional structure for the instrument. Model 2 is acceptable but less effective, while Model 3, with a single-factor structure, shows a substantially poorer fit.

Table 3 presents the results of factor loadings, internal consistency (α and H), average variance extracted (AVE), the square root of AVE (√AVE), and correlations between the dimensions of the NWVS-I scale. A table within which we provide Pearson correlations for all items is available in the Supplementary Material.

The factor loadings of the items range from 0.53 (INC 2) to 0.89 (INC 1), indicating that most items exhibit good levels of saturation on their respective factors.

The results indicate that Cronbach’s alpha values ranged from 0.61 (REL—relating) to 0.82 (MEA—meaning), suggesting acceptable reliability. Additionally, Hancock’s H values also ranged from 0.61 (REL—relating) to 0.82 (MEA—meaning), confirming the construct’s reliability in the confirmatory factor analysis (CFA) framework.

The AVE ranges from 0.44 (REL) to 0.69 (MEA). According to convergent validity criteria, an AVE value of ≥0.50 is generally considered acceptable. Although some dimensions (e.g., REL and FLE) have slightly lower values, most dimensions meet the criterion.

The √AVE values are all greater than the correlations with other dimensions, confirming the discriminant validity of the dimensions.

Significant correlations (*p* < 0.05) between dimensions range from low values (e.g., REL and RFC = 0.13) to moderate–high values (e.g., CSR and MEA = 0.55).

Statistically significant differences emerged between males and females. Specifically, males reported significantly higher scores than females for the autonomy dimension; females reported significantly higher scores than males for the following dimensions: corporate social responsibility, inclusion, security, participation, and meaning (a table reporting means, standard deviations, significance levels, and Cohen’s d values are provided in the Supplementary Material). The effects of these differences were medium (ranging from 0.21 to 0.50).

Table 4 presents the correlations between the 14 dimensions of the NWVS-I scale and the four value dimensions of Schwartz’s theory: openness to change (OPEN), self-enhancement (ENHA), self-transcendence (TRAN), and conservatism (CONS).

Openness to change (OPEN) is positively and moderately correlated with the NWVS-I’s subdimensions of autonomy (AUT), stimulation (STI), and readiness for change (RFC).

Self-enhancement (ENHA) is positively and moderately correlated with money (MON).

Self-transcendence (TRAN) shows strong or moderate correlations with corporate social responsibility (CSR), meaning (MEA), participation (PAR), inclusion (INC), clarity (CLA), security (SEC), and development (DEV), and a negative low correlation with money (MON).

Conservatism (CONS) is positively and moderately correlated with security (SEC), clarity (CLA), and relating (REL).

Table 5 presents the results of gender invariance testing. Based on the threshold values proposed in the literature (ΔCFI ≤ 0.01 and ΔRMSEA ≤ 0.015), configural invariance and metric invariance can be confirmed. In the first case, the model fit is good; in the second case, the RMSEA and CFI values remain unchanged, and both ΔRMSEA and ΔCFI support metric invariance.

Regarding scalar invariance, the RMSEA value remains stable, meeting the invariance criteria. However, the CFI value decreases, with a ΔCFI of 0.013, which exceeds the 0.01 threshold. Although the RMSEA indicates a good model fit, exceeding the ΔCFI threshold suggests a potential violation of scalar invariance.

Finally, the ΔCFI and ΔRMSEA values for residual invariance indicate a clear violation of residual invariance.

## 4. Discussion

This study validates the Italian form of the New Work Values Scale (NWVS; Stiglbauer et al., 2022), providing a significant contribution to the literature on work-related values. Overall, the results confirm the good psychometric validity of the instrument, with some critical points that warrant further exploration.

Regarding the factorial structure, we tested three different factorial models: the 14-factor model, the 14-factor model with three high-order factors, and a single-factor model. Among these, the 14-factor model showed the best fit to the data, supporting the theoretical hypothesis that work values are distinct and specific constructs. This finding is consistent with the original model proposed by Stiglbauer et al. (2022), reinforcing the idea that work values are better conceptualized as a set of articulated dimensions rather than a general construct or as aggregated categories.

Discriminant validity was generally satisfactory, with some exceptions. Specifically, the flexibility and relating dimensions showed AVE values slightly below 0.50, suggesting that the variance explained by the items in these dimensions is lower than desirable (Hair et al., 2010). This issue could be attributed to the inherently heterogeneous nature of these constructs. For instance, flexibility might encompass aspects that vary significantly depending on the work context or individual experiences, making it challenging to represent coherence through the items. Similarly, the idea of relating, which pertains to the ability to establish positive workplace relationships, might be influenced by personal or cultural factors not fully captured by the NWVS items. Despite these limitations, the discriminant validity of the instrument was robust. In all cases, the square root of the AVE for each dimension exceeded the correlations between dimensions, indicating that each factor measures a distinct construct (Fornell & Larcker, 1981; Koufteros, 1999).

Furthermore, findings from the concurrent validity study indicated that the dimensions of the NWVS-I were significantly related to Schwartz’s Portrait Values Questionnaire (PVQ; Schwartz et al., 2001). The correlations between *openness to change* and *autonomy*, *stimulation*, and *readiness for change* reflect an emphasis on exploration, change, and independence. The dimension of self-enhancement correlates with the *money* dimension of the NWVS-I scale, aligning with the idea that financial gain and success are central to self-affirmation values. Self-transcendence is strongly correlated with the values associated with altruism and collective well-being, such as *corporate social responsibility*, *meaning*, and *inclusion*. Finally, the correlation of *security*, *clarity*, and *relating* with *conservatism* reflects the need for stability, order, and harmonious relationships. This analysis highlights how Schwartz’s value dimensions are instrumental to understanding the underlying structure of the NWVS-I scale values.

The gender invariance analyses confirm that the structure of the model is similar across gender groups, meaning that the items measure the same constructs (factors) for both groups. Additionally, men and women interpret the scale items in a similar manner, allowing for comparisons of latent factor relationships across genders. However, men and women might interpret some items differently and may have gender-specific response patterns to certain items, regardless of their actual level in the construct being measured. These results limit the ability to directly compare factor means between groups, but it is still possible to compare correlations and relationships between factors for men and women. Overall, while its use for direct mean comparisons requires caution, the NWVS-I demonstrates robust structural and metric properties across genders.

The results also point to broader cultural and contextual elements at work. Dimensions like flexibility and job security take on particular significance in the Italian socio-economic context, marked by regional disparities and a labor market ever more touched by the growing centrality of work–life balance (Bentivogli et al., 2019; Camussi et al., 2021), especially following the increase in occupations that require smart working (Zammitti et al., 2022). Additionally, changes in values such as clarity and stimulation, accelerated by the effects of the COVID-19 pandemic, further demonstrate the effectiveness of the NWVS in representing the fluid nature of work orientations (Ramskogler, 2022).

## 5. Implications and Future Directions

These findings provide empirical support for the use of the NWVS-I in Italian research on work values and careers. Even if a work–life balance and job security remain strong, traditional priorities for Italian workers, including values concerned with autonomy, career development, and employer branding, are becoming more prominent. This shift underscores the challenge for organizations to align human resources planning with changing employee expectations, thereby creating work cultures that balance stability and flexibility (in line with the post-modern culture). Although derived from a relatively modest sample, these findings point to the potential avenues for future research, including the examination of trends in the industry and the long-term development of work values in the context of economic and technological developments. However, some limitations should be acknowledged. Specifically, the fact that each dimension is measured by only two items raises potential concerns regarding the identification of single-factor models and the robustness of factor determination. Some dimensions might need item modification in order to improve both internal consistency and explained variance. For example, future research could attempt to add new items to dimensions with lower AVE values in order to increase their general representativeness.

Further research in other work settings could also help to determine whether the problems found are due to cultural influences or specific to the sector. The complexity and variety within some of the dimensions also point out the need to develop both theoretical and operational definitions.

The NWVS-I presents significant practical considerations for organizations aiming to synchronize their human resource management practices with the values of their employees. Aspects such as corporate social responsibility, inclusion, and flexibility are key to improving employer branding strategies, building organizational commitment, and attracting talent, especially among young people (Stiglbauer et al., 2022; Buzzao & Rizzi, 2024). Moreover, understanding employees’ preferences regarding career advancement versus job security could allow organizations to design more targeted development programs and retention strategies, adequately addressing regional differences and labor-market concerns (Camussi et al., 2021) as well as onboarding processes. Future research could also investigate the relation of NWVS dimensions to organizational outcomes, such as job satisfaction, performance, and turnover intention. Longitudinal studies of how work values change over time and among generations would be especially insightful in response to changes in society, such as growing remote work and economic instability. Furthermore, applying the NWVS to diverse industries and cultural settings would test its generalizability and increase its cross-cultural validity.

## 6. Conclusions

In summary, the NWVS-I is a valid and useful tool for investigating work values in Italy. Although some critical points emerged, these did not significantly compromise the overall reliability of the instrument. These findings provide further support for the use of the NWVS-I in future studies to investigate more deeply the role of work value within contemporary workplaces and its relationship with other psychological constructs.

The NWVS-I responds to a lack in the availability of concise and multidimensional measures of work values within the Italian setting while, at the same time, providing a framework that answers some core organizational needs. This allows organizations to identify specific value congruences and discrepancies for the enhancement of the well-being, engagement, and productivity of their employees. With this multidimensional framework, as workplace environments evolve, the NWVS-I brings a substantive view to understanding and supporting the dynamic interaction of individual values within professional settings in order to foster both the sustainable growth of the organization and employee satisfaction.

## Figures and Tables

**Table 1:** Descriptive statistics of New Work Values Scale—Italian form (NWVS-I).

Item	M	SD	Skewness	Kurtosis
RFC 1	4.14	0.84	−0.60	−0.29
RFC 2	4.15	0.86	−0.67	−0.30
CSR 1	4.45	0.80	−1.60	2.78
CSR 2	4.46	0.79	−1.47	1.75
INC 1	4.61	0.70	−1.76	2.36
INC 2	4.31	0.85	−0.98	0.03
SEC 1	4.56	0.76	−1.76	2.67
SEC 2	4.55	0.69	−1.57	2.46
PAR 1	4.39	0.75	−1.04	0.57
PAR 2	4.55	0.68	−1.39	1.33
CLA 1	4.60	0.64	−1.55	1.88
CLA 2	4.48	0.68	−1.09	0.49
FLE 1	4.48	0.67	−1.08	0.57
FLE 2	4.76	0.50	−1.92	2.88
MON 1	3.46	0.90	−0.25	0.11
MON 2	3.37	1.10	−0.30	−0.48
CAR 1	4.30	0.81	−1.17	1.50
CAR 2	4.27	0.94	−1.25	1.13
DEV 1	4.45	0.73	−1.23	1.28
DEV 2	4.55	0.69	−1.54	2.10
STI 1	3.96	0.88	−0.57	−0.04
STI 2	3.90	0.93	−0.60	−0.01
AUT 1	3.76	0.98	−0.37	−0.44
AUT 2	3.78	0.94	−0.24	−0.72
MEA 1	4.52	0.75	−1.55	1.84
MEA 2	4.46	0.79	−1.47	1.91
REL 1	4.07	0.91	−0.73	−0.01
REL 2	3.88	0.98	−0.67	0.04

Note. RFC = readiness for change; CSR = corporate social responsibility; INC = inclusion; SEC = security; PAR = participation; CLA = clarity; FLE = flexibility; MON = money; CAR = career; DEV = development; STI = stimulation; AUT = autonomy; MEA = meaning; REL = relating.

**Table 2:** Confirmatory factor analysis results.

	χ^2^	df	χ^2^/df	CFI	RMSEA (C.I. 90%)	SRMR	AIC	∆ AIC
Model 1 (14 factors)	422.851	259	1.63	0.959	0.040 (0.033–0.047)	0.035	716.851	–
Model 2 (14 factors, 3 high-order factors)	763.359	334	2.29	0.891	0.057 (0.052–0.062)	0.064	907.359	190.508
Model 3 (single factor)	2092.925	350	5.980	0.559	0.112 (0.108–0.117)	0.094	2204.925	1297.566

Note. χ^2^ = chi-squared; df = degrees of freedom; CFI = Comparative Fit Index; RMSEA = Root Mean Square Error of Approximation; SRMR = Standardized Root Mean Square Residual; AIC = Akaike Information Criterion.

**Table 3:** Factor loadings, internal consistency (α), average variance extracted (AVE), square root of AVE (√AVE), and correlations between the dimensions of the NWVS-I scale.

Item	Factor Loading	α	H	AVE	√AVE	RFC	CSR	INC	SEC	PAR	CLA	FLE	MON	CAR	DEV	STI	AUT	MEA	REL
**RFC 1**	0.75	0.68	0.68	0.52	0.72	1													
**RFC 2**	0.68																		
**CSR 1**	0.65	0.67	0.68	0.52	0.72	0.41 **	1												
**CSR 2**	0.78																		
**INC 1**	0.89	0.63	0.69	0.54	0.73	0.32 **	0.57 **	1											
**INC 2**	0.53																		
**SEC 1**	0.82	0.80	0.80	0.67	0.82	0.19 **	0.33 **	0.34 **	1										
**SEC 2**	0.82																		
**PAR 1**	0.67	0.67	0.67	0.51	0.71	0.44 **	0.45 **	0.37 **	0.37 **	1									
**PAR 2**	0.75																		
**CLA 1**	0.84	0.77	0.78	0.63	0.79	0.32 **	0.36 **	0.32 **	0.40 **	0.41 **	1								
**CLA 2**	0.75																		
**FLE 1**	0.73	0.61	0.63	0.46	0.68	0.32 **	0.40 **	0.47 **	0.36 **	0.53 **	0.46 **	1							
**FLE 2**	0.63																		
**MON 1**	0.77	0.74	0.75	0.60	0.77	0.02	−0.51	−0.04	0.24 **	−0.01	0.05	0.03	1						
**MON 2**	0.78																		
**CAR 1**	0.72	0.67	0.67	0.51	0.71	0.25 **	0.18 **	0.23 **	0.31 **	0.24 **	0.34 **	0.25 **	0.24 **	1					
**CAR 2**	0.70																		
**DEV 1**	0.78	0.77	0.77	0.63	0.79	0.33 **	0.42 **	0.32 **	0.32 **	0.39 **	0.42 **	0.34 **	−0.01	0.57 **	1				
**DEV 2**	0.81																		
**STI 1**	0.60	0.65	0.66	0.50	0.71	0.28 **	0.16 **	0.18 **	0.11 *	0.26 **	0.25 **	0.13 *	0.05	0.36 **	0.40 **	1			
**STI 2**	0.80																		
**AUT 1**	0.81	0.81	0.81	0.68	0.82	0.22 **	0.10	0.07	0.01	0.16 **	0.15 **	0.15 **	0.22 **	0.15 **	0.15 **	0.21 **	1		
**AUT 2**	0.84																		
**MEA 1**	0.81	0.82	0.82	0.69	0.83	0.27 **	0.55 **	0.37 **	0.29 **	0.42 **	0.43 **	0.36 **	−0.06	0.27 **	0.51 **	0.24 **	0.15 **	1	
**MEA 2**	0.85																		
**REL 1**	0.66	0.61	0.61	0.44	0.66	0.13 *	0.16 **	0.15 **	0.23 **	0.28 **	0.20 **	0.22 **	0.22 **	0.28 **	0.28 **	0.22 **	0.10 *	0.27 **	1
**REL 2**	0.67																		

Note. α = internal consistency; H = Hancock’s H; AVE = average variance extracted; RFC = readiness for change; CSR = corporate social responsibility; INC = inclusion; SEC = security; PAR = participation; CLA = clarity; FLE = flexibility; MON = money; CAR = career; DEV = development; STI = stimulation; AUT = autonomy; MEA = meaning; REL = relating; * *p* < 0.05; ** *p* < 0.01.

**Table 4:** Correlations between the 14 dimensions of the NWVS-I scale and the value dimensions of Schwartz’s theory.

	OPEN	ENHA	TRAN	CONS
RFC	**0.33** **	0.08	**0.32** **	0.09
CSR	0.22 **	−0.06	**0.55** **	0.25 **
INC	0.14 **	−0.04	**0.40** **	0.22 **
SEC	0.13 *	0.03	**0.30** **	**0.34** **
PAR	0.25 **	0.06	**0.44** **	0.24 **
CLA	0.20 **	0.07	**0.35** **	**0.31** **
FLE	0.20 **	−0.02	**0.32** **	0.21 **
MON	0.20 **	**0.33** **	−0.12 *	0.19 **
CAR	0.27 **	0.25 **	0.23 **	0.23 **
DEV	0.26 **	0.06	**0.47** **	0.28 **
STI	**0.34** **	0.25 **	0.27 **	0.23 **
AUT	**0.38** **	0.25 **	0.10 *	0.18 **
MEA	0.20 **	−0.02	**0.57** **	0.28 **
REL	0.25 **	0.12 *	0.29 **	**0.32** **

Note. * *p* < 0.05; ** *p* < 0.01; RFC = readiness for change; CSR = corporate social responsibility; INC = inclusion; SEC = security; PAR = participation; CLA = clarity; FLE = flexibility; MON = money; CAR = career; DEV = development; STI = stimulation; AUT = autonomy; MEA = meaning; REL = relating; OPEN = openness to change; CONS = conservatism; ENHA = self-enhancement; TRAN = self-transcendence. The highest correlations are indicated in bold.

**Table 5:** Goodness-of-fit statistics for the test of gender invariance.

	χ^2^	df	RMSEA	ΔRMSEA	CFI	ΔCFI
Configural	767.301	518	0.035	-	0.938	-
Metric	782.159	532	0.035	0.000	0.938	0.000
Scalar	937.299	665	0.035	0.000	0.925	0.013
Residual	1107.996	665	0.041	0.006	0.889	0.036

Note. χ^2^ = chi-squared; df = degrees of freedom; CFI = Comparative Fit Index; RMSEA = Root Mean Square Error of Approximation.

## Data Availability

The data presented in this study are available upon request from the corresponding author due to privacy reasons.

## Supplementary Materials

The following supporting information can be downloaded at https://www.mdpi.com/article/10.3390/ejihpe15030028/s1. Item-category frequencies for all items; Pearson correlations for all items; Gender differences across all dimensions of NWVS-I.

## Appendix A

New Work Values Scale—Italian form (NWVS-I)

**Work Value**	**Item**	**English/Italian**
Readiness for Change	RFC1	A company should dare to sometimes try something new [Un’azienda dovrebbe osare nel provare qualcosa di nuovo ogni tanto]
	RFC2	Innovative companies particularly appeal to me [Le aziende innovative mi attraggono particolarmente]
Corporate Social Responsibility	CSR1	I would rather work for a company that helps make the world a better place [Preferirei lavorare per un’azienda che aiuta a rendere il mondo un posto migliore]
	CSR2	Sustainability should be a key issue for all companies [La sostenibilità dovrebbe essere una questione chiave per tutte le aziende]
Inclusion	INC1	A company should put money into accessibility so that no one is discriminated against [Un’azienda dovrebbe investire nell’accessibilità in modo che nessuno venga discriminato]
	INC2	It is important to me that the proportion of women working there is taken into account in a company [Per me è importante che in un’azienda si tenga conto della percentuale di donne che vi lavorano]
Security	SEC1	A secure job is very important to me [Un lavoro sicuro è molto importante per me]
	SEC2	If I were to look for a new job, job security would be very important to me [Se dovessi cercare un nuovo lavoro, la sicurezza del lavoro sarebbe molto importante per me]
Participation	PAR1	In a good company, all employees should have the opportunity to contribute ideas [In una buona azienda, tutti i dipendenti dovrebbero avere l’opportunità di contribuire con le proprie idee]
	PAR2	If employees are asked for their opinion and can have a say, that is a form of appreciation [Se ai dipendenti viene chiesta la loro opinione e possono avere voce in merito, questa è una forma di apprezzamento]
Clarity	CLA1	A company should have clear structures [Un’azienda dovrebbe avere una struttura organizzativa (linee guida o regole aziendali) ben definita]
	CLA2	I think employers should set clear rules that can be used as a guide [Penso che i datori di lavoro dovrebbero stabilire regole chiare che possano essere utilizzate come guida]
Flexibility	FLE1	I expect an employer to be understanding and flexible for unforeseeable private events [Mi aspetto che un datore di lavoro sia comprensivo e flessibile nel caso di imprevisti personali]
	FLE2	Work–life balance is very important to me [L’equilibrio vita-lavoro è molto importante per me]
Money	MON1	A high salary is the most important thing to me [Uno stipendio elevato è la cosa più importante per me]
	MON2	I draw the greatest motivation for my work from a high salary [Traggo la maggiore motivazione nel mio lavoro da uno stipendio elevato]
Career	CAR1	Opportunities for advancement motivate me [Le opportunità di avanzamento mi motivano]
	CAR2	I want to make a career in my job [Voglio fare carriera nel mio lavoro]
Development	DEV1	Further training is important to me [La formazione continua è importante per me]
	DEV2	In my job, I always want to develop myself and my knowledge [Nel mio lavoro, voglio sempre sviluppare me stesso e la mia conoscenza]
Stimulation	STI1	There has to be something going on at my work before I feel good [Ho bisogno di un certo livello di attività al lavoro per sentirmi a mio agio]
	STI2	I really enjoy a job when I am always faced with new challenges [Mi diverto davvero in un lavoro quando mi trovo sempre di fronte a nuove sfide]
Autonomy	AUT1	I prefer to design my work completely freely according to my own specifications [Preferisco progettare il mio lavoro in totale libertà secondo le mie indicazioni]
	AUT2	In my job, it is important for me to be able to decide for myself when I do which activity [Nel mio lavoro, è importante per me poter decidere autonomamente quando fare quale attività]
Meaning	MEA1	I want to do something good for others with my work [Voglio fare qualcosa di buono per gli altri con il mio lavoro]
	MEA2	With my work, I want to contribute to making the world a better place [Con il mio lavoro, voglio contribuire a rendere il mondo un posto migliore]
Relating	REL1	It is important for me to be friends with my colleagues [È importante per me essere amico dei miei colleghi]
	REL2	Regular social events alongside work are a sign of good company [Gli abituali eventi sociali con i colleghi organizzati dall’azienda sono segno di una buona organizzazione]

## References

[B1-ejihpe-15-00028] Anglim J., Molloy K., Dunlop P. D., Albrecht S. L., Lievens F., Marty A. (2022). Values assessment for personnel selection: Comparing job applicants to non-applicants. European Journal of Work and Organizational Psychology.

[B2-ejihpe-15-00028] Arieli S., Sagiv L., Roccas S. (2020). Values at work: The impact of personal values in organisations. Applied Psychology.

[B3-ejihpe-15-00028] Backhaus K., Tikoo S. (2004). Conceptualizing and researching employer branding. Career Development International.

[B4-ejihpe-15-00028] Bauman Z. (2013). Liquid modernity.

[B5-ejihpe-15-00028] Beaton D. E., Bombardier C., Guillemin F., Ferraz M. B. (2000). Guidelines for the process of cross-cultural adaptation of self-report measures. Spine.

[B6-ejihpe-15-00028] Bentivogli C., Ferraresi T., Monti P., Paniccia R., Rosignoli S. (2019). Italian regions in global value chains: An input-output approach. Politica Economica.

[B7-ejihpe-15-00028] Bentler P. M. (1990). Comparative fit indexes in structural models. Psychological Bulletin.

[B8-ejihpe-15-00028] Bharadwaj S. (2023). CSR employer branding, organisational identification, person–organisation fit and employee retention: A dual mediation model. Journal of Economic and Administrative Sciences.

[B9-ejihpe-15-00028] Blustein D. L., Ali S. R., Flores L. Y. (2019). Vocational psychology: Expanding the vision and enhancing the impact. The Counseling Psychologist.

[B10-ejihpe-15-00028] Bosch M. J., Hernández T., Las Heras Maestro M., Chinchilla Albiol N., Grau Grau M. (2020). A closer look to millennials in Chile: How they perceive the new i-deal worker. The new ideal worker. Contributions to management science.

[B11-ejihpe-15-00028] Brown T. A. (2015). Confirmatory factor analysis for applied research.

[B12-ejihpe-15-00028] Burnham K. P., Anderson D. R. (2004). Multimodel inference: Understanding AIC and BIC in model selection. Sociological Methods & Research.

[B13-ejihpe-15-00028] Busque-Carrier M., Ratelle C. F., Le Corff Y. (2021). Work values and job satisfaction: The mediating role of basic psychological needs at work. Journal of Career Development.

[B14-ejihpe-15-00028] Buzzao G., Rizzi F. (2024). Who is CSR for in employer branding? Insights on employer branding strategies across industries, educational backgrounds and career styles. Corporate Social Responsibility and Environmental Management.

[B15-ejihpe-15-00028] Byrne B. M. (2010). Structural equation modeling with AMOS: Basic concepts, applications, and programming.

[B16-ejihpe-15-00028] Camussi S. A., Maccarrone V., Aimone Gigio L. (2021). Changes in the employment structure and in job quality in Italy: A national and regional analysis.

[B17-ejihpe-15-00028] Capanna C., Vecchione M., Schwartz S. H. (2005). La misura dei valori. Un contributo alla validazione del Portrait Values Questionnaire su un campione italiano. Bollettino di Psicologia Applicata.

[B18-ejihpe-15-00028] Cohen J. (1988). Statistical power analysis for the behavioral sciences.

[B19-ejihpe-15-00028] Cohen J. (2013). Statistical power analysis for the behavioral sciences.

[B20-ejihpe-15-00028] Cronbach L. J. (1951). Coefficient alpha and the internal structure of tests. Psychometrika.

[B22-ejihpe-15-00028] Deci E. L., Olafsen A. H., Ryan R. M. (2017). Self-determination theory in work organizations: The state of a science. Annual Review of Organizational Psychology and Organizational Behavior.

[B21-ejihpe-15-00028] Deci E. L., Ryan R. M. (2000). The “what” and “why” of goal pursuits: Human needs and the self-determination of behavior. Psychological Inquiry.

[B23-ejihpe-15-00028] Dose J. J. (1997). Work values: An integrative framework and illustrative application to organizational socialization. Journal of Occupational and Organizational Psychology.

[B24-ejihpe-15-00028] Flaxman P. E., Bond F. W., Livheim F. (2013). The mindful and effective employee: An acceptance and commitment therapy training manual for improving well-being and performance.

[B25-ejihpe-15-00028] Fornell C., Larcker D. F. (1981). Evaluating structural equation models with unoberservable variables and measurement error. Journal of Marketing Research.

[B26-ejihpe-15-00028] Forner V. W., Jones M., Berry Y., Eidenfalk J. (2020). Motivating workers: How leaders apply self-determination theory in organizations. Organization Management Journal.

[B27-ejihpe-15-00028] Fossen R. J. S., Vredenburgh D. J. (2014). Exploring differences in work’s meaning: An investigation of individual attributes associated with work orientations. Journal of Behavioral and Applied Management.

[B28-ejihpe-15-00028] Fraccaroli F., Zaniboni S., Truxillo D. M. (2024). Challenges in the new economy: A new era for work design. Annual Review of Organizational Psychology and Organizational Behavior.

[B29-ejihpe-15-00028] Hair J. F., Black W. C., Babin B. J., Anderson R. E. (2010). Multivariate data analysis: Pearson College division.

[B30-ejihpe-15-00028] Hancock G. R., Mueller R. O. (2001). Rethinking construct reliability within latent variable systems. Structural Equation Modeling: Present and Future.

[B31-ejihpe-15-00028] Höge T. (2011). Perceived flexibility requirements at work and the entreployee-work-orientation: Concept and measurement. Journal Psychologie des Alltagshandelns/Psychology of Everyday Activity.

[B32-ejihpe-15-00028] Hu L.-t., Bentler P. M. (1999). Cutoff criteria for fit indexes in covariance structure analysis: Conventional criteria versus new alternatives. Structural Equation Modeling.

[B33-ejihpe-15-00028] Jöreskog K. G., Sörbom D. (1993). LISREL 8: Structural equation modeling with the SIMPLIS command language.

[B34-ejihpe-15-00028] Kasih E., Ruslaini R. (2024). The impact of diversity and inclusion initiatives on organizational performance.

[B35-ejihpe-15-00028] Kim M., Kim J. (2020). Corporate social responsibility, employee engagement, well-being and the task performance of frontline employees. Manag. Decision.

[B36-ejihpe-15-00028] Kline R. B. (2016). Principles and practice of structural equation modeling.

[B37-ejihpe-15-00028] Koufteros X. A. (1999). Testing a model of pull production: A paradigm for manufacturing research using structural equation modeling. Journal of Operations Management.

[B38-ejihpe-15-00028] Laskin A. V., Kresic K. (2021). Inclusion as a component of CSR and a brand connection strategy. Public relations for social responsibility: Affirming DEI commitment with action.

[B39-ejihpe-15-00028] Magnano P., Zammitti A., Lodi E. (2024). Positive resources to promote well-being in educational and work contexts and in career trajectories [Topical collection]. European Journal of Investigation in Health, Psychology and Education.

[B40-ejihpe-15-00028] Magnusson K. (2021). Interpreting Cohen’s d effect size: An interactive visualization (Version 2.5.1). R Psychologist.

[B41-ejihpe-15-00028] Mauk S. S., Halek Y. A. (2022). Analysis of the effect of corporate social responsibility (CSR) on risk, performance, and company value (good management theory based testing). Jurnal Manajemen Bisnis Dan Organisasi.

[B42-ejihpe-15-00028] Nord W. R., Brief A. P., Atieh J. M., Doherty E. M., Staw B. M., Cummings L. L. (1988). Work values and the conduct of organizational behavior. Research in organizational behavior.

[B43-ejihpe-15-00028] Pitacho L. A., Palma P., Correia P. (2019). Work orientation: Dimensionality and internal model. Análise Psicológica.

[B44-ejihpe-15-00028] Putnick D. L., Bornstein M. H. (2016). Measurement invariance conventions and reporting: The state of the art and future directions for psychological research. Developmental Review.

[B45-ejihpe-15-00028] Ramskogler P. (2022). Feeling the heat? Assessing labor shortages in the euro area. SURF Policy Brief.

[B46-ejihpe-15-00028] Reis G. G., Braga B. M. (2016). Employer attractiveness from a generation perspective: Implications for employer branding. Rausp Management Journal.

[B47-ejihpe-15-00028] Reis I., Sousa M. J., Dionísio A. (2021). Employer branding as a talent management tool: A systematic literature revision. Sustainability.

[B48-ejihpe-15-00028] Rigby C. S., Ryan R. M. (2018). Self-determination theory in human resource development: New directions and practical considerations. Advances in Developing Human Resources.

[B49-ejihpe-15-00028] Robitzsch A. (2023). To check or not to check? A comment on the contemporary psychometrics (ConPsy) checklist for the analysis of questionnaire items. European Journal of Investigation in Health, Psychology and Education.

[B50-ejihpe-15-00028] Ros M., Schwartz S. H., Surkiss S. (1999). Basic individual values, work values, and the meaning of work. Applied Psychology: An International Review.

[B51-ejihpe-15-00028] Rounds J. B., Henly G. A., Dawis R. V., Lofquist L. H., Weiss D. J. (1981). Manual for the Minnesota importance questionnaire: A measure of vocational needs and values.

[B52-ejihpe-15-00028] Schwartz S., Zanna M. P. (1992). Universals in the content and structure of values: Theoretical advances and empirical tests in 20 countries. Advances in experimental social psychology.

[B53-ejihpe-15-00028] Schwartz S. H. (1999). A theory of cultural values and some implications for work. Applied Psychology: An International Review.

[B54-ejihpe-15-00028] Schwartz S. H., Melech G., Lehmann A., Burgess S., Harris M., Owens V. (2001). Extending the cross-cultural validity of the theory of basic human values with a different method of measurement. Journal of Cross-Cultural Psychology.

[B55-ejihpe-15-00028] Soper D. S. (2023). A-priori Sample Size Calculator for Structural Equation Models. https://www.danielsoper.com/statcalc.

[B56-ejihpe-15-00028] Sousa R. C. (2021). Work values for generations Y and Z stricto sensu accounting students. Contextus-Contemporary Journal of Economics and Management.

[B57-ejihpe-15-00028] Steiger J. H. (1990). Structural model evaluation and modification: An interval estimation approach. Multivariate Behavioral Research.

[B58-ejihpe-15-00028] Stiglbauer B., Penz M., Batinic B. (2022). Work values across generations: Development of the New Work Values Scale (NWVS) and examination of generational differences. Frontiers in Psychology.

[B59-ejihpe-15-00028] Super D. E. (1970). Work values inventory manual.

[B60-ejihpe-15-00028] Tabachnick B., Fidell L. (2013). Using multivariate statistics.

[B61-ejihpe-15-00028] Theurer C. P., Tumasjan A., Welpe I. M., Lievens F. (2018). Employer branding: A brand equity-based literature review and research agenda. International Journal of Management Reviews.

[B62-ejihpe-15-00028] Trivellas P., Rafailidis A., Polychroniou P., Dekoulou P. (2019). Corporate social responsibility (CSR) and its internal consequences on job performance. International Journal of Quality and Service Sciences.

[B63-ejihpe-15-00028] Willner T., Lipshits-Braziler Y., Gati I. (2019). Construction and initial validation of the work orientation questionnaire. Journal of Career Assessment.

[B64-ejihpe-15-00028] Zammitti A., Russo A., Magnano P., Guarnera M. (2022). Work engagement as a moderating factor between positive attitude toward smart working and job and life satisfaction. European Journal of Investigation in Health, Psychology and Education.

